# Feasibility of Left Bundle Branch Area Pacing via a Persistent Left Superior Vena Cava in a Patient with Repaired Ventricular Septal Defect

**DOI:** 10.19102/icrm.2026.17024

**Published:** 2026-02-15

**Authors:** Serhat Kesriklioglu, Ahmet Taha Sahin, Khaled Alwaled, Abdulhadi Elkadiki, Ahmet Lutfu Sertdemir, Enes Elvin Gul

**Affiliations:** 1 Department of Cardiology, Necmettin Erbakan School of Medicine, Konya, Turkey; 2 Department of Cardiology, Kahta State Hospital, Adiyaman, Turkey; 3 Metiga Military Hospital, Metiga, Libya; 4 Venezia Hospital, Benghazi, Libya

**Keywords:** Conduction system pacing, left bundle branch area pacing, persistent left superior vena cava, ventricular septal defect

## Abstract

Persistent left superior vena cava (PLSVC) is the most common systemic venous anomaly and may complicate cardiac device implantation, particularly in patients with a history of congenital heart surgery. We report the case of a 16-year-old girl with a surgically repaired ventricular septal defect who developed complete heart block, necessitating conduction system pacing (CSP). This case highlights the feasibility of CSP in the presence of PLSVC and prior congenital heart surgery.

## Introduction

Persistent left superior vena cava (PLSVC) is the most common thoracic venous anomaly, found in 0.3%–0.5% of the general population and more frequently in patients with congenital heart disease.^1^ Although often clinically silent, its presence may complicate cardiac interventions, particularly device implantation, electrophysiological procedures, and surgical repair.^2^

Pacemaker implantation via a PLSVC has been described, yet reports of advanced pacing strategies such as conduction system pacing (CSP) in this anatomy remain rare.^3^ In patients operated for ventricular septal defect (VSD), achieving optimal left bundle branch pacing can be technically demanding, and the coexistence of a PLSVC further compounds the procedural complexity. We present the case of an adolescent patient with a history of VSD repair who developed complete atrioventricular block (AVB) requiring pacemaker implantation.

This case highlights the interplay between congenital venous anomalies, prior surgical repair, and the growing role of CSP in complex anatomical settings.

## Case presentation

A 16-year-old girl with a history of surgically repaired VSD and complete AVB was referred for permanent pacemaker implantation. A 12-lead resting electrocardiogram (ECG) revealed complete AVB with junctional escape rhythm at 40 bpm. Transthoracic echocardiography showed preserved left ventricular function (left ventricular ejection fraction [LVEF], 50%) and right ventricular (RV) dilatation. After obtaining consent, she was taken to the laboratory for pacemaker implantation.

**Figure 1:**
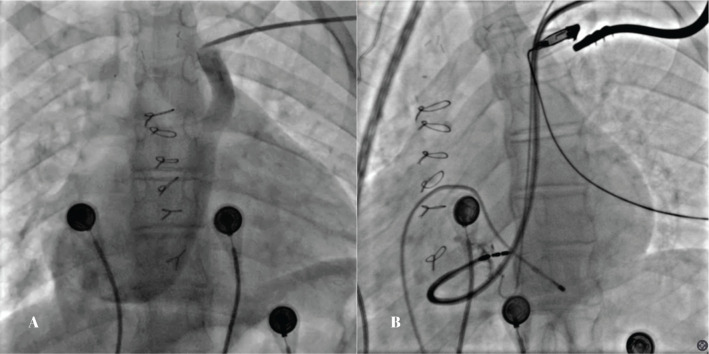
**A:** Contrast venography illustrating a persistent left superior vena cava. **B:** Intraprocedural fluoroscopic image during left bundle branch area pacing, demonstrating adequate penetration of the pacing lead into the interventricular septum.

**Figure 2:**
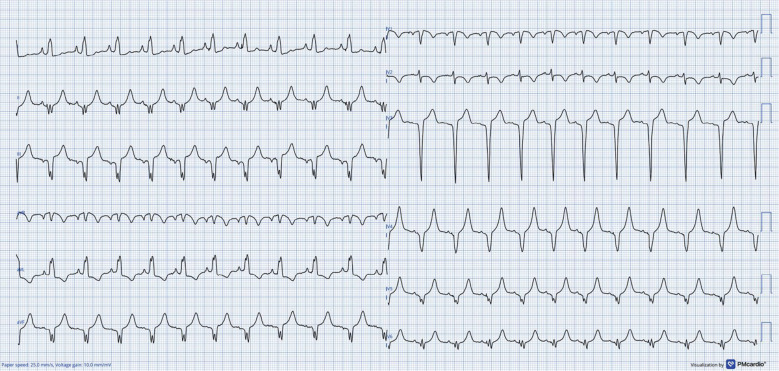
Twelve-lead electrocardiogram obtained after the procedure.

During device implantation, a PLSVC with no antegrade flow toward the right-sided superior vena cava was found. A dedicated sheath for the left bundle branch implantation (Selectra 3D 55/42; Biotronik, Berlin, Germany) was introduced and advanced into the right ventricle. Contrast was injected in the right anterior oblique 30° view to assess the distance from the tricuspid annulus. Then, a Solia S 60 lead (Biotronik) was used to penetrate the septum. Lead penetration was performed under continuous pacing in the left anterior oblique 30° view. Due to anatomical variation and the presence of a VSD patch, placement of the lead into the septum was challenging. After a few attempts, we were able to penetrate the lead into the septum with satisfactory sensing and pacing parameters **(Figures 1A and 1B)**. Unfortunately, despite deep penetration, there was no r′ in V1, and transition was not present. A septogram revealed that both the ring and tip were inside the septum. Due to normal LVEF and a challenging anatomy, we opted to accept this outcome. The final paced QRS complex showed a QS pattern with a QRS duration of 130 ms, which was in favor of deep septal pacing. Although the final paced ECG showed a narrow QRS width, due to altered conduction property secondary to the VSD patch, it is not always possible to capture the conduction system. After stabilizing the RV lead in the septum, the sheath slitted, with no evidence of dislodgement. The right atrial lead was placed in the lateral wall with good pacing parameters. A dual-chamber pacemaker device (Enticos DR MRI; Biotronik) was used. The total procedure and fluoroscopy times were 95 and 21 min, respectively. The final paced ECG revealed a QRS duration of 130 ms **(Figure 2)**.

The post-procedural course was uneventful, and the patient was discharged in good clinical condition with a stable rhythm and scheduled follow-up in the cardiac rhythm and device clinic.

## Discussion

Surgical closure of perimembranous VSD carries an inherent risk of conduction disturbances because of the close proximity of the defect to the atrioventricular node and bundle of His.^4^ Postoperative complete heart block occurs in 1%–4% of cases, and, while most conduction abnormalities resolve within 7–10 days, a subset remains persistent and requires permanent pacemaker implantation. Younger age, lower body weight, longer cross-clamp time, and increased surgical complexity have been identified as risk factors.^5^

PLSVC is the most common thoracic venous anomaly, present in up to 0.5% of the general population and more frequently among patients with congenital heart disease. The most common cardiac anomalies accompanying PLSVC are single ventricle, atrioventricular septal defect, and tetralogy of Fallot.^6^

The presence of PLSVC can complicate venous access, pacemaker implantation, and lead positioning. Reports of left bundle branch area pacing (LBBAP) in the setting of PLSVC suggest that, although technically demanding, the procedure is feasible with careful adaptation of delivery tools and techniques. In recent years, several reports have demonstrated the feasibility of CSP, including LBBAP, in the presence of PLSVC, though the anatomical course of the vein frequently requires modified implantation strategies or right-sided approaches.^7–9^ Current delivery sheaths are primarily designed for left-sided implantation, which may necessitate additional technical modifications to perform LBBAP via the PLSVC. Bulava et al. reported that, even in the absence of bridging veins, LBBAP can be successfully achieved via the coronary sinus in patients with PLSVC.^9^ Recent reports have highlighted that, in patients with PLSVC, CSP strategies, such as LBBAP-optimized cardiac resynchronization therapy (CRT), can serve as an effective alternative when conventional CRT implantation is not feasible.^8^ In our patient, the coexistence of repaired VSD along with PLSVC made the procedure more challenging.

Vlach et al. emphasized that, in patients with complex congenital heart disease and unconventional venous anatomy, LBBAP remains feasible when careful preprocedural planning and creative modifications of delivery catheters are employed.^10^ In the presence of a PLSVC, some operators prefer right-sided pacemaker implantation to simplify lead positioning and reduce procedural complexity. Unfortunately, in our case, the pocket was opened prior to venography. Therefore, we opted to carry on with the left-sided implantation. Our experience highlights that, with appropriate sheath manipulation and procedural planning, successful septal lead deployment is achievable even through unconventional venous anatomy. This case emphasizes the need for individualized procedural planning, awareness of anatomical variations, and the integration of advanced pacing strategies to optimize outcomes. Furthermore, it adds to the limited but growing body of evidence that CSP can be safely and effectively performed in patients with PLSVC when appropriate adjustments are made, thereby broadening the applicability of physiological pacing in challenging anatomical scenarios.

## Conclusion

This case illustrates that CSP can be successfully performed in the presence of a PLSVC and a history of VSD repair. Recognition of such anatomical variations and the use of tailored procedural strategies are essential to ensure safe and effective long-term rhythm management in this complex population.
